# Reproductive performance of beef cattle with ovarian hypofunction and repeat breeding in Jepara Regency, Central Java, Indonesia

**DOI:** 10.14202/vetworld.2021.784-787

**Published:** 2021-03-27

**Authors:** Aldi Salman, Surya Agus Prihatno, Bambang Sumiarto

**Affiliations:** 1 Department of Livestock and Animal Health, Central Java Province, Indonesia; 2 Department of Reproduction and Obstetric, Faculty of Veterinary Medicine, Universitas Gadjah Mada, Indonesia; 3 Department of Veterinary Public Health, Faculty of Veterinary Medicine, Universitas Gadjah Mada, Indonesia

**Keywords:** beef cattle, Jepara Regency, ovarian hypofunction, repeat breeding, reproductive disorders, reproductive performance

## Abstract

**Background and Aim:**

Reproductive disorders keep the beef cattle population in Jepara Regency, Central Java, Indonesia, from increasing. Ovarian hypofunction and repeat breeding are the most common reproductive disorders, leading to large economic losses for traditional breeders. However, the impact of poor reproductive performance among traditional breeders is not well-known. This study aimed to investigate the reproductive performance of beef cattle with ovarian hypofunction and repeat breeding in Jepara Regency.

**Materials and Methods:**

We determined cattle’s reproductive status by rectal examination and anamnesis, assessing reproductive performance in 28 cows with repeat breeding and 27 cows with ovarian hypofunction. The following parameters were measured: Postpartum estrous (PPE), days open (DO), service per conception (S/C), and calving interval (CI). The data came from livestock records from animal recording cards, iSIKHNAS, and estimated births from the insemination date that produced a pregnancy.

**Results:**

In beef cattle diagnosed with ovarian hypofunction, S/C, PPE, DO, and CI were 1.28, 257 days, 265 days, and 18 months, respectively. The length of CI caused by long PPE may be due to improper nutrition and calf weaning delays. In beef cattle with repeat breeding, S/C, PPE, DO, and CI were 4.15, 106 days, 210 days, and 16 months, respectively. The length of CI was caused by long DO due to pregnancy failure at the first estrus.

**Conclusion:**

Hypofunction and repeat breeding reduce the reproductive performance of beef cattle in the Jepara Regency. The cows’ health conditions pre- and postpartum can be optimized by providing high-quality feed to enhance reproductive performance.

## Introduction

The increasing need for animal protein led to increased beef cattle populations in Central Java. The livestock system in Central Java relies on traditional rural landless integrated agricultural systems, especially rice plants [1]. One of the main factors that ­prevent increases in the beef cattle population is reproductive disorders. The prevalence of reproductive disorders in beef cattle in Indonesia, which is between 11% and 57%, is still quite high. Ovarian hypofunction and repeat breeding are the most common reproductive disorders found in Jepara Regency. More than 40% of cattle were diagnosed with reproductive disorders in 2017 due to ovarian hypofunction and repeat breeding [2].

Ovarian hypofunction or inactive ovaries is characterized by no growth of follicles and corpus luteum in the ovaries, which is clinically manifested as anestrus. During rectal palpation, the surface of the ovary is smooth and slick. Poor health and long-term food shortages cause ovarian hypofunction, leading to ovarian atrophy [3]. On the other hand, repeat breeding, which is characterized by failure to conceive after three or more regularly spaced artificial inseminations (AI), often occurs in cows with no clinical abnormalities. The cows will come into heat within 17-24 days after AI. This failure can be caused by fertilization failure or embryo death [4].

Both ovarian hypofunction and repeat breeding cause massive economic losses for traditional breeders. Increased calving intervals (CIs) cause economic losses, increased insemination requirements, increased veterinary examination and treatment costs, and additional maintenance costs. Unfortunately, the impact of poor reproductive performance, especially among traditional breeders, is not well understood.

This study aimed to investigate the reproductive performance of beef cattle with ovarian hypofunction and repeat breeding in Jepara Regency.

## Materials and Methods

### Ethical approval

Research Ethics Committee of the Faculty of Veterinary Medicine, Universitas Gadjah Mada (UGM) [Approval no.: 0004/EC-FKH/Eks./2020] reviewed and approved this study.

### Study period and location

This research was conducted for 8 months, from October 2019 to May 2020. The research was conducted on local farm among traditional breeders at Jepara Regency, one of the largest beef cattle breeding areas in Central Java.

### Test animals

The research subjects included Ongole cross cattle that birthed <5 times were under 8 years of age or had normally functioning reproductive organs. The animal was a heifer or postpartum. We examined animals in confined conditions; they were restrained either in a box or by a rope. Rectal palpation and anamnesis were performed to determine the reproductive status. Repeat breeding was diagnosed in 28 cows, and ovarian hypofunction was diagnosed in 27 cows. Reproductive activities were followed in these animals until pregnancy.

### Data management and analysis

Reproductive performance was assessed by measuring postpartum estrous (PPE), days open (DO), service per conception (S/C), and CI. Reproductive performance was tabulated and analyzed by processing livestock record data and analyzing the data based on reproduction parameters. The data were obtained from animal record cards, iSIKHNAS, and estimated births from the date of successful insemination. Data are presented as averages with standard deviations.

## Results

We measured reproductive performance in beef cattle diagnosed with ovarian hypofunction S/C, PPE, DO, and CI were 1.28, 257 days, 265 days, and 18 months, respectively, while beef cattle with repeat breeding were 4.15, 106 days, 210 days, and 16 months, as shown in Table-1. Both diagnoses showed decrease reproductive performance, mainly prolonged calving interval.

**Table-1:** Reproductive performance of beef cattle with ovarian hypofunction and repeat breeding in Jepara Regency.

Diagnosis	No. of cattle	S/C	PPE	DO	CI
Ovarian hypofunction	27	1.28	257.84±68.86	265.24±64.04	18.28±2.11
Repeat breeding	28	4.15	106.93±17.88	210.81±25.32	16.04±0.88

PPE=Postpartum estrous, DO=Days open, S/C=Service per conception, CI=Calving interval

## Discussion

The average optimum S/C score in beef cattle with ovarian hypofunction is 1.28. This optimal S/C occurs because most of the cows diagnosed with ovarian hypofunction are in the lactation phase. After calf weaning, improvement in body condition occurs, and cows return to estrous. A negative energy balance occurs when nutrition intake does not meet the higher energy requirements, mainly in breastfeeding cows. The condition of the uterus and ovaries correlates with improved body condition after weaning. The follicular development wave reaches a dominant follicular diameter sufficient to increase estradiol concentration and triggers a surge in pre-ovulatory gonadotropins [5,6].

A negative energy balance manifests in other reproductive disorders also. During the early postpartum period, a weaker body condition causes energy deficiency for hormone synthesis and secretion to ovulate a follicle and sustain an early developing embryo. Repeat breeding occurs when ovaries produce low-quality oocytes and embryos or when the uterus is unable to sustain the pregnancy. Cows with repeat breeding had an average S/C score of 4.15, which is much higher than the standard S/C for Indonesian traditional breeders of 1.5-2. Traditional cattle breeding systems are prone to reproductive failure due to poor selection of breeds and low-quality feed. Cows fed with low nutritional feed are very vulnerable to reproductive disorders [7,8].

Postpartum estrus is the interval after calving until the appearance of the first estrous sign. In cows with ovarian hypofunction, PPE occurred at 257.84±70.28 days. After calving, cows experience a temporary period of infertility known as postpartum anestrus. The target PPE is between 50 and 60 days after calving. Extension of the anestrus period in beef cattle in the Jepara Regency may be related to lactation status and low nutritional intake, causing negative energy balance. After calf weaning, the energy balance improves, and good quality dominant follicles and oocytes are produced [7]. PPE can be extended by silent ovulation if there is a negative energy balance over a long period. Ovulation without estrous symptoms can occur for up to three cycles [9]. In cows with repeat breeding, PPE occurred at 106.93±17.88 days. Negative energy balance seems to be the underlying cause for the dysfunctions, including prolonged standing estrus and delayed ovulation, leading to fertilization failure. Even with good signs of estrus, the cow will seek mating 21 days later [10].

**Figure-1:**
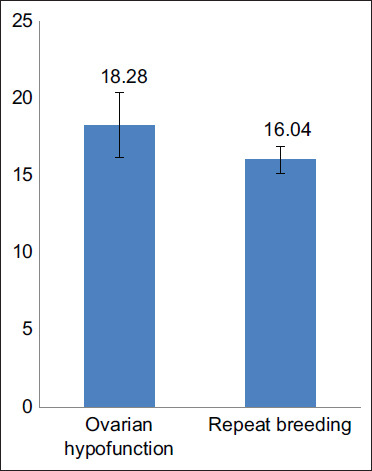
Average days open in beef cattle with ovarian hypofunction and repeat breeding in Jepara Regency.

Beef cattle with ovarian hypofunction had an average DO of 265.24 days (Figure-1). Thus, successful insemination could occur 265 days after the last calving. Factors that affect DO are different from PPE. Variables affecting DO include inseminator skills, AI time, and the breeders’ knowledge in detecting estrus [8]. In cows with repeat breeding, DO occurred at 210.81±25.32 days. When the DO was longer than PPE, farmers incurred greater costs, including maintenance, breeding, and treatment costs. Furthermore, the application of AI can lead to pregnancy failures due to fertilization failure and embryo death, resulting in longer DO [11,12].

**Figure-2:**
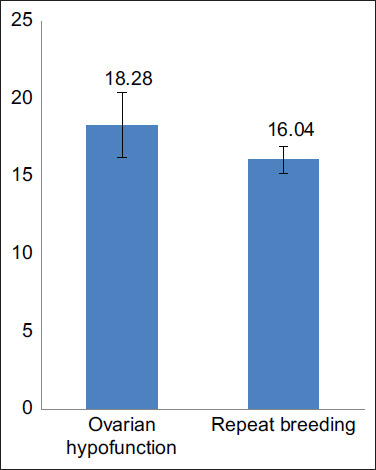
Average calving intervals in beef cattle with ovarian hypofunction and repeat breeding in Jepara Regency.

CI is calculated based on the distance between the last recorded birth and the estimated day of birth from the last successful mating date. CIs in cattle with ovarian hypofunction and repeat breeding were 18.28±2.15 months and 16.04±0.88 months (Figure-2). Longer CIs were caused by long postpartum estrus due to delays in weaning calves. Furthermore, pregnancy failure at the first estrus can prolong DO [12].

Variations in DO and CI in beef cattle with ovarian hypofunction were greater than cattle with repeat breeding. The increased variations were due to calf weaning age variations, which are a major factor in the return of ovarian activity in cows with ovarian hypofunction. Shortening the CI will optimize the number of births in a cow’s lifetime, providing greater benefits. All efforts to optimize reproduction aim to shorten the CI to obtain greater profit. There is an association between the CI and the number of births; CI becomes longer for every consecutive birth [13,14].

## Conclusion and Recommendations

S/C, PPE, DO, and CI were 1.28, 257 days, 265 days, and 18 months, respectively, in beef cattle diagnosed with ovarian hypofunction and 4.15, 106 days, 210 days, and 16 months, respectively, in beef cattle with repeat breeding. Longer CIs in cattle with ovarian hypofunction occur due to the length of PPE. In cattle with repeat breeding, the longer CI is due to high S/C.

The body condition scores reflect the amount of metabolic energy stored as subcutaneous fat and muscle in beef cattle. Optimizing the nutritional intake during pre- and postpartum periods, adding supplemental diets, and reducing periparturition problems is expected to enhance beef cows’ reproduction performance.

## Authors’ Contributions

AS, SAP, and BS conceived the study design. AS and SAP identified the reproductive status and collected individual cow data. SAP and BS helped in the acquisition of data and drafted the manuscript. All authors read and approved the final manuscript.
